# ECLip-the European consortium on lipodystrophies: an update

**DOI:** 10.1186/1750-1172-10-S2-O32

**Published:** 2015-11-11

**Authors:** David Araújo-Vilar

**Affiliations:** 1 Member of ECLip Executive Board, Division of Endocrinology and Nutrition, University Clinical Hospital of Santiago de Compostela, Santiago de Compostela, Spain

## 

The European Consortium of Lipodystrophies (ECLip) is a network of relevant clinical and basic-science research groups in Europe involved in investigation of Lipodystrophic Syndromes (LS).

The goal of this Consortium is to enable intensive and effective collaboration among the various high-quality European research groups in order to promote the free exchange of ideas and information concerning research and clinical care among LS researchers. The principal benefit will be the advancement of patient care. It will also promote the public understanding of LS and its consequences in affected individuals. On the other hand, ECLip will lead to further growth and inclusion of novel aspects of LS research, making European investigators the leaders in this important but still poorly explored research area. Likewise, ECLip will try to give visibility and recognition of LS in society and among policy makers, and will help the promotion of advocacy groups in Europe and worldwide.

To date, ECLip is formed by 38 research groups coming from 15 European countries.

The ECLip website (http://www.european-lipodystrophies.org) provides information on all groups involved in the consortium, with information about the researchers, the main research lines of each group, their clinical and basic research facilities, and contact details.

